# Time Series AI Model for Acute Kidney Injury Detection Based on a Multicenter Distributed Research Network: Development and Verification Study

**DOI:** 10.2196/47693

**Published:** 2024-07-05

**Authors:** Suncheol Heo, Eun-Ae Kang, Jae Yong Yu, Hae Reong Kim, Suehyun Lee, Kwangsoo Kim, Yul Hwangbo, Rae Woong Park, Hyunah Shin, Kyeongmin Ryu, Chungsoo Kim, Hyojung Jung, Yebin Chegal, Jae-Hyun Lee, Yu Rang Park

**Affiliations:** 1 Department of Biomedical System Informatics, Yonsei University College of Medicine, Seoul, Republic of Korea; 2 Medical Informatics Collaborative Unit, Department of Research Affairs, Yonsei University College of Medicine, Seoul, Republic of Korea; 3 Department of Computer Engineering, Gachon University, Seongnam, Republic of Korea; 4 Transdisciplinary Department of Medicine & Advanced Technology, Seoul National University Hospital, Seoul, Republic of Korea; 5 Healthcare AI Team, National Cancer Center, Goyang, Republic of Korea; 6 Department of Biomedical Informatics, Ajou University School of Medicine, Suwon, Republic of Korea; 7 Healthcare Data Science Center, Konyang University Hospital, Daejeon, Republic of Korea; 8 Department of Biomedical Sciences, Ajou University Graduate School of Medicine, Suwon, Republic of Korea; 9 Department of Statistics, Korea University, Seoul, Republic of Korea; 10 Division of Allergy and Immunology, Department of Internal Medicine, Yonsei University College of Medicine, Seoul, Republic of Korea; 11 Institute of Allergy, Yonsei University College of Medicine, Seoul, Republic of Korea

**Keywords:** adverse drug reaction, real world data, multicenter study, distributed research network, common data model, time series AI, time series, artificial intelligence, machine learning, adverse reaction, adverse reactions, detect, detection, toxic, toxicity, renal, kidney, nephrology, pharmaceutical, pharmacology, pharmacy, pharmaceutics

## Abstract

**Background:**

Acute kidney injury (AKI) is a marker of clinical deterioration and renal toxicity. While there are many studies offering prediction models for the early detection of AKI, those predicting AKI occurrence using distributed research network (DRN)–based time series data are rare.

**Objective:**

In this study, we aimed to detect the early occurrence of AKI by applying an interpretable long short-term memory (LSTM)–based model to hospital electronic health record (EHR)–based time series data in patients who took nephrotoxic drugs using a DRN.

**Methods:**

We conducted a multi-institutional retrospective cohort study of data from 6 hospitals using a DRN. For each institution, a patient-based data set was constructed using 5 drugs for AKI, and an interpretable multivariable LSTM (IMV-LSTM) model was used for training. This study used propensity score matching to mitigate differences in demographics and clinical characteristics. Additionally, the temporal attention values of the AKI prediction model’s contribution variables were demonstrated for each institution and drug, with differences in highly important feature distributions between the case and control data confirmed using 1-way ANOVA.

**Results:**

This study analyzed 8643 and 31,012 patients with and without AKI, respectively, across 6 hospitals. When analyzing the distribution of AKI onset, vancomycin showed an earlier onset (median 12, IQR 5-25 days), and acyclovir was the slowest compared to the other drugs (median 23, IQR 10-41 days). Our temporal deep learning model for AKI prediction performed well for most drugs. Acyclovir had the highest average area under the receiver operating characteristic curve score per drug (0.94), followed by acetaminophen (0.93), vancomycin (0.92), naproxen (0.90), and celecoxib (0.89). Based on the temporal attention values of the variables in the AKI prediction model, verified lymphocytes and calcvancomycin ium had the highest attention, whereas lymphocytes, albumin, and hemoglobin tended to decrease over time, and urine pH and prothrombin time tended to increase.

**Conclusions:**

Early surveillance of AKI outbreaks can be achieved by applying an IMV-LSTM based on time series data through an EHR-based DRN. This approach can help identify risk factors and enable early detection of adverse drug reactions when prescribing drugs that cause renal toxicity before AKI occurs.

## Introduction

Acute kidney injury (AKI) is associated with a mortality rate of 40%‐70% in hospitalized patients who develop AKI and causes significant kidney damage even after recovery, leading to dialysis, longer hospital stays, and increased costs of care [1-4]. Early detection of AKI increases the likelihood of AKI prevention, associated morbidity, and costs [5]. As no specific treatment can reverse AKI and the recognition of patients at risk of AKI before diagnosis contributes to better clinical outcomes than treatment after AKI occurs [6], early detection of AKI is essential for prompt therapeutic intervention.

Several studies have attempted to predict AKI occurrence. With the increasing availability of clinical databases, they developed models to predict the occurrence of AKI using electronic health records (EHRs) [7-18]. Although these studies used EHRs, the number of patients in the patient population was small because they focused on specific patients, such as surgical patients, patients with sepsis, and older adults. There have also been a number of studies using artificial intelligence (AI) models to predict AKI. Although attempts have been made to predict the occurrence of AKI early, few models have provided clear rationales and explanations [19-21]. Therefore, time series data analysis is required for AKI prediction models to reflect the temporal information between variables [22]. Time series analysis for AKI is necessary because the length of time each patient stays in a hospital or intensive care unit can differ from person to person, and the frequency of measurements can vary from values that are measured continuously (eg, blood pressure) to laboratory values that are measured on an as-needed basis. Recently, an interpretable multivariable long short-term memory (IMV-LSTM) method for considering time series data has been published [23]; however, little research has been conducted on this method.

To address these issues, this study aimed to apply and validate a multicenter-based explainable time series AI model for predicting the occurrence of AKI caused by specific nephrotoxic drugs in 6 hospitals in South Korea by using a large clinical database with a common data model (CDM) through a distributed research network (DRN).

## Methods

### Ethical Considerations

This study was approved by the institutional review committees of Severance Hospital (SH; 4-2021-1209), Gangnam Severance Hospital (GSH; 3-2021-0005), Konyang University Hospital (KYUH; 2021-10-003-001), Ajou University Hospital (AJUH; AJIRB-MED-MDB-21‐676), Seoul National University Cancer Hospital (SNUH; E-2207-151-1342), and the National Cancer Center (NCC; 2022-0184). All retrospective data were anonymized and appropriate measures were taken to protect participant information.

### Study Design

**Figure 1.**
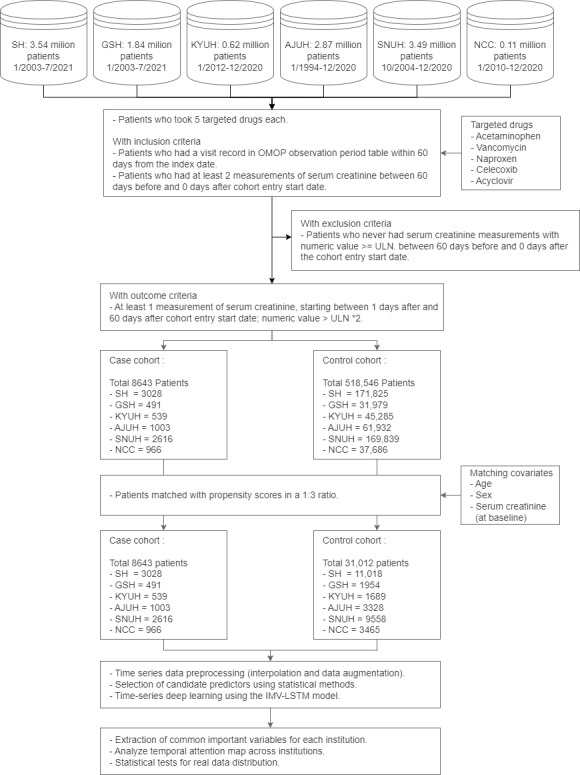
Overall flowchart for predicting acute kidney injury events. AJUH: Ajou University Hospital; GSH: Gangnam Severance Hospital; IMV-LSTM: interpretable multivariable long short-term memory; KYUH: Konyang University Hospital; NCC: National Cancer Center; OMOP: Observational Medical Outcomes Partnership; SH: Severance Hospital; SNUH: Seoul National University Cancer Hospital; ULN, upper limit of normal.

This retrospective observational cohort study analyzed the EHRs from 6 hospitals in South Korea between 1994 and 2021 to predict AKI. The EHRs were converted to OMOP-CDM (Observational Medical Outcomes Partnership Common Data Model) version 5.3.1. The 6 hospitals were SH, GSH, KYUH, AJUH, SNUH, and the NCC. An overall diagram of the cohort composition is provided in Figure 1.

In our cohorts, we adopted criteria based on serum creatinine (SCr) to define AKI according to the Kidney Disease: Improving Global Outcomes (KDIGO) Clinical Practice Guidelines and the previously defined AKI classification stages mapped in the “injury” category [24-26]. The criterion is an increase in the SCr level to 2 times the baseline value. As an alternative to the baseline SCr levels, we defined the upper limit of normal (ULN) value of SCr as 1.2 mg/dl [27].

The targeted drugs were selected from 5 medications associated with a high risk of AKI according to the US Food and Drug Administration (FDA) and previous studies: acetaminophen; vancomycin; 2 nonsteroidal anti-inflammatory drugs (NSAIDs), naproxen and celecoxib; and 1 antiviral drug, acyclovir [21,22].

The inclusion criteria were as follows: (1) the target drugs were administered, (2) the patient had a visit record of at least 30 days prior to the observation period, and (3) the patient underwent at least 2 SCr tests during the preobservation period (0‐60 days before the study). The exclusion criteria were as follows: (1) the patient had at least 1 SCr test outside the ULN value in the preobservation period. Participants were divided into case and control cohorts based on whether they met previously defined AKI criteria for 60 days after taking the first medicine. The observation period refers to the time range before and after the initial medication intake for each patient within the cohort. The cohort definitions were created using ATLAS, a web-based tool (Observational Health Data Sciences and Informatics), and are available as JSON files on GitHub [28].

To adjust for differences between cases and controls to reduce the effect of confounding variables, we used propensity score matching (PSM). Covariates included were age, sex, and SCr value at baseline. We normalized the covariates by applying standard scaling to ensure consistent dimensions across variables. A propensity score for each patient was generated by logistic regression. Patients were matched in a 1:3 ratio using a K-nearest neighbor (K-NN) algorithm using the Python *scikit-learn* library.

### Candidate Predictors for Time Series

Candidate predictors were extracted from several key domains within the CDM containing per-patient observational data using structured query language tools in Python. Age and sex were used in the *person* domain, clinical laboratory tests in the *measurement* domain, medications in the *drug exposure* domain, diagnostic records in the *condition occurrence* domain, and surgical/procedure records in the *procedure occurrence* domain. Lab tests were treated as continuous variables, while other medications, conditions, and procedures were treated as binary variables. Statistical methods were used to select the variables. To identify predictors, we tested the statistical significance of the difference between the enrollment time of the cohort and the onset date of AKI using a 2-tailed paired *t* test and the McNemar test for continuous and dichotomous variables, respectively. To create a time series table, the candidate variables were pivoted into columns and dates were placed into rows. Missing values were handled in the following ways: forward fill for laboratory tests and diagnoses and zero fill for medications and treatments. The window size for predictions used 4-week sequence data and was processed by shifting the data of the prediction cycle by 2 weeks.

### AKI Prediction Modeling

LSTM models based on recurrence have been designed to process time series data [29]. Attention-based LSTM models were initially proposed for learning words and the relationships between words in natural language processing [30,31] and later evolved into a key component of deep learning, becoming one of the methods used to provide interpretations, including importance scores for predicted outcomes.

As an advanced LSTM model, we used the IMV-LSTM module for the learning model, which is a multivariate LSTM neural network for the prediction and interpretation of multivariate time series [23]. This model improves on the LSTM-attention model, which can predict variable importance using multivariate inputs to configure variable-wise hidden states and mix both temporal and variable levels of attention for improved interpretability. The model was trained for 200 epochs with a batch size of 64 and a learning rate of 1e-3. An Adam optimizer was used with early stopping after 20 epochs. The data set was divided into training, test, and validation sets at a 6:2:2 ratio. Prediction performance was evaluated using the area under the receiver operating characteristic curve (AUROC) value. Additionally, we used the accuracy, precision, *F*_1_-score, and area under the precision-recall curve (AUPRC) to ensure robustness for unbalanced data.

In this study, AKI prediction models were created for each hospital and drug. Each model had a different selection of candidate variables. To interpret the predictors in each model, variable- and temporal-wise attention scores were extracted from all trained models. These scores were then aggregated by calculating the overall temporal attention score, which was obtained by taking the weighted average of the temporal attention value over the attention value for each predictor variable. The resulting scores were plotted as heat maps for interpretation.

### Statistical Analysis

We used statistical packages based on Python and R (R Project for Statistical Computing) for the statistical analysis. First, to compare the AKI and non-AKI groups, we calculated significance using the *χ*^2^ test for categorical variables and an independent-sample 2-tailed *t* test for continuous variables. Second, to identify differences in the pattern of AKI occurrence between cohorts and drugs, a histogram was plotted for patients in each cohort from the date of cohort entry (the first day of administration of the target drug) to the date of AKI occurrence. Differences between drugs were analyzed using an independent-sample2-tailed *t* test. Third, we compared the distribution of the aggregated temporal attention scores with the actual trained data with box plots of the data for 4 weeks at 1-week intervals. A repeated ANOVA test was performed to identify temporal differences.

## Results

### Demographic and Clinical Characteristics

The demographics of the 31,012 patients without AKI and the 8643 patients with AKI across the 6 hospitals after PSM are shown in Table 1.

**Table 1.** Demonstration and clinical characteristics of patients with and without AKI across 6 hospitals after propensity score matching. The *P* values were obtained by conducting a 2-sample *t* test to compare the means for cases and controls.

		Case group							Control group							*P* value
		SH^a^ (n=3028)	GSH^b^ (n=491)	KYUH^c^ (n=539)	AJUH^d^ (n=1003)	SNUH^e^ (n=2616)	NCC^f^ (n=966)	Total (n=8643)	SH (n=11,018)	GSH (n=1689)	KYUH (n=1954)	AJOU (n=3328)	SNUH (n=9558)	NCC (n=3465)	Total (n=31,012)	
Age (years), mean (SD)		61.83 (15.23)	62.6 (15.04)	67.91 (13.64)	59.52 (15.79)	57.89 (16.49	60.55 (12.24)	60.65 (15.5)	61.14 (15.37)	62.26 (14.81)	67.71 (13.55)	59.67 (15.77)	57.46 (16.61)	60.14 (12.71)	60.21 (15.61)	.02
**Gender, n (%)**																
	Male	1965 (64.89)	286 (58.25)	360 (66.79)	628 (62.61)	1652 (63.15)	569 (58.9)	5460 (63.17)	7092 (64.37)	985 (58.32)	950 (48.62)	1765 (53.03)	4602 (48.15)	2018 (58.24)	17,412 (56.15)	<.001
	Female	1063 (35.11)	205 (41.75)	179 (33.21)	375 (37.39)	964 (36.85)	397 (41.1)	3183 (36.83)	3926 (35.63)	704 (41.68)	1004 (51.38)	1563 (46.97)	4956 (51.85)	1447 (41.76)	13,600 (43.85)	<.001
Sepsis, n (%)		325 (10.73)	50 (10.18)	7 (1.3)	139 (13.86)	40 (1.53)	1 (0.1)	562 (6.5)	373 (3.39)	37 (2.19)	21 (1.07)	128 (3.85)	41 (0.43)	1 (0.03)	601 (1.94)	<.001
Diabetes mellitus, n (%)		915 (30.22)	117 (23.83)	65 (12.06)	223 (22.23)	300 (11.47)	59 (6.11)	1679 (19.43)	2462 (22.35)	266 (15.75)	195 (9.98)	593 (17.82)	836 (8.75)	167 (4.82)	4519 (14.57)	<.001
Chronic kidney disease, n (%)		142 (4.69)	3 (0.61)	24 (4.45)	13 (1.3)	18 (0.69)	2 (0.21)	202 (2.34)	271 (2.46)	8 (0.47)	51 (2.61)	11 (0.33)	40 (0.42)	3 (0.09)	384 (1.24)	<.001
Chronic liver disease, n (%)		462 (15.26)	56 (11.41)	52 (9.65)	139 (13.86)	506 (19.34)	16 (1.66)	1231 (14.24)	766 (6.95)	82 (4.85)	71 (3.63)	126 (3.79)	759 (7.94)	42 (1.21)	1846 (5.95)	<.001
Hypoalbuminemia, n (%)		7 (0.23)	2 (0.41)	0	0	0	0	9 (0.1)	9 (0.08)	2 (0.12)	0	0	0	0	11 (0.04)	.01
Hypotension, n (%)		12 (0.4)	4 (0.81)	0	1 (0.1)	4 (0.15)	0	21 (0.24)	30 (0.27)	3 (0.18)	0	4 (0.12)	28 (0.29)	0	65 (0.21)	.56
Hypertension, n (%)		1462 (48.28)	179 (36.46)	77 (14.29)	351 (35)	264 (10.09)	71 (7.35)	2404 (27.81)	4178 (37.92)	455 (26.94)	360 (18.42)	1143 (34.34)	924 (9.67)	229 (6.61)	7289 (23.5)	<.001
Neoplasm (active cancers), n (%)		2112 (69.75)	320 (65.17)	255 (47.31)	652 (65)	2081 (79.55)	581 (60.14)	6001 (69.43)	5512 (50.03)	531 (31.44)	417 (21.34)	1282 (38.52)	4450 (46.56)	2470 (71.28)	14,662 (47.28)	<.001
Heart failure, n (%)		266 (8.78)	13 (2.65)	34 (6.31)	33 (3.29)	62 (2.37)	6 (0.62)	414 (4.79)	629 (5.71)	20 (1.18)	105 (5.37)	60 (1.8)	127 (1.33)	10 (0.29)	951 (3.07)	<.001
Obesity, n (%)		3 (0.1)	2 (0.41)	0	2 (0.2)	3 (0.11)	0	10 (0.12)	36 (0.33)	3 (0.18)	4 (0.2)	10 (0.3)	50 (0.52)	0	103 (0.33)	<.001
Peripheral vascular disease, n (%)		25 (0.83)	9 (1.83)	19 (3.53)	18 (1.79)	10 (0.38)	0	81 (0.94)	51 (0.46)	26 (1.54)	69 (3.53)	35 (1.05)	37 (0.39)	2 (0.06)	220 (0.71)	.03
Liver dysfunction, n (%)		62 (2.05)	2 (0.41)	2 (0.37)	40 (3.99)	31 (1.19)	2 (0.21)	139 (1.61)	71 (0.64)	4 (0.24)	25 (1.28)	46 (1.38)	59 (0.62)	3 (0.09)	208 (0.67)	<.001
Anemia, n (%)		541 (17.87)	25 (5.09)	35 (6.49)	89 (8.87)	154 (5.89)	4 (0.41)	848 (9.81)	903 (8.2)	53 (3.14)	68 (3.48)	176 (5.29)	282 (2.95)	7 (0.2)	1489 (4.8)	<.001
Prior kidney surgery, n (%)		0	0	1 (0.19)	3 (0.3)	14 (0.54)	0	18 (0.21)	0	0	0	0	11 (0.12)	1 (0.03)	12 (0.04)	<.001
**Laboratory values (before medication), mean (SD)**																
	Serum creatinine (mg/dL)	0.46 (0.74)	0.79 (0.23)	0.99 (0.37)	0.87 (0.37)	0.87 (0.47)	1.01 (0.29)	0.75 (0.59)	0.44 (0.51)	0.76 (0.19)	0.98 (0.22)	0.84 (0.2)	0.83 (0.18)	0.98 (0.16)	0.72 (0.4)	<.001
	Glucose (mg/dL)	126.89 (58.33)	132.1 (52.11)	150.75 (82.36)	187.0 (23.0)	121.48 (48.26)	125.85 (50.05)	126.93 (56.28)	121.72 (55.7)	124.3 (49.43)	140.11 (78.81)	151.5 (39.5)	115.65 (40.5)	120.88 (46.23)	120.95 (51.85)	<.001
	Potassium (mmol/L)	3.92 (0.6)	4.21 (0.47)	4.14 (0.6)	4.17 (0.55)	4.22 (0.5)	4.29 (0.56)	4.11 (0.57)	3.93 (0.55)	4.22 (0.4)	4.09 (0.54)	4.15 (0.46)	4.22 (0.43)	4.28 (0.52)	4.11 (0.51)	.48
	Sodium (mmol/L)	138.6 (4.16)	137.27 (4.36)	136.49 (4.61)	138.48 (4.23)	138.46 (3.96)	138.31 (3.67)	138.3 (4.14)	139.44 (3.75)	138.31 (3.58)	137.74 (3.73)	139.27 (3.85)	139.71 (3.23)	138.96 (3.37)	139.28 (3.6)	<.001
	BUN (blood urea nitrogen) (mg/dL)	17.83 (9.66)	15.44 (6.58)	17.67 (8.87)	14.68 (6.36)	14.72 (7.61)	15.55 (6.13)	16.13 (8.28)	16.82 (7.48)	15.78 (5.86)	17.19 (7.62)	14.9 (6.41)	14.67 (5.14)	14.63 (4.96)	15.67 (6.47)	<.001

a SH: Severance Hospital.

b GSH: Gangnam Severance Hospital.

c KYUH: Konyang University Hospital.

d AJUH: Ajou University Hospital.

e SNUH: Seoul National University Cancer Hospital.

f NCC: National Cancer Center.

By hospital, the cohort consisted of 14,046 patients from SH, 2180 from GSH, 2493 from KYUH, 4331 from AJUH, 13,174 from SNUH, and 4431 from NCC after matching the propensity scores. Propensity matching was performed using age, sex, and SCr levels at baseline. As for the changes in covariates, the difference in mean age decreased from 6.39 (60.65 – 54.26) to 0.44 (60.65 – 60.21) years, the difference in male ratio decreased from 16.49% (63.17% – 46.68%) to 7.02% (63.17% – 56.15%), and the difference in SCr at baseline decreased from 0.14 mg/dL (0.71 – 0.58 mg/dL) to 0.03 mg/dL (0.71 – 0.67 mg/dL) (Table S2 in Multimedia Appendix 1). There were still statistically significant differences in PSM age (60.66, SD 15.86 vs 60.22, SD 15.94 years; *P*=.03), gender (63.17% vs 56.15% male; *P*<.001), and SCr at baseline (0.71, SD 0.61 vs 0.68, SD 0.41 mg/dL; *P*<.001). Patients who developed AKI had more severe neoplasms (ie, active cancers; 70.6% vs 44.26%; *P*<.001) and chronic liver disease (15.83% vs 6.55%; *P*<.001). Moreover, the analysis revealed the following differences: sepsis (6.5% vs 1.94%; *P*<.001), diabetes mellitus (19.43% vs 14.57%; *P*<.001), hypertension (27.81% vs 23.5%; *P*<.001), anemia (9.81% vs 4.8%; *P*<.001), and heart failure (4.79% vs 3.07%; *P*<.001). There was no significant difference between hypotension (0.24% vs 0.21%; *P*=.56), potassium (4.11, SD 0.57 vs 4.11, SD 0.51 mmol/L; *P*=.48), or renal artery stenosis (0.07% vs 0.03%; *P*=.13). Hypoalbuminemia, obesity, peripheral vascular disease, renal artery stenosis, liver dysfunction, and prior kidney surgery had low incidence rates (<2%).

### Distribution of Adverse Drug Events

**Figure 2.**
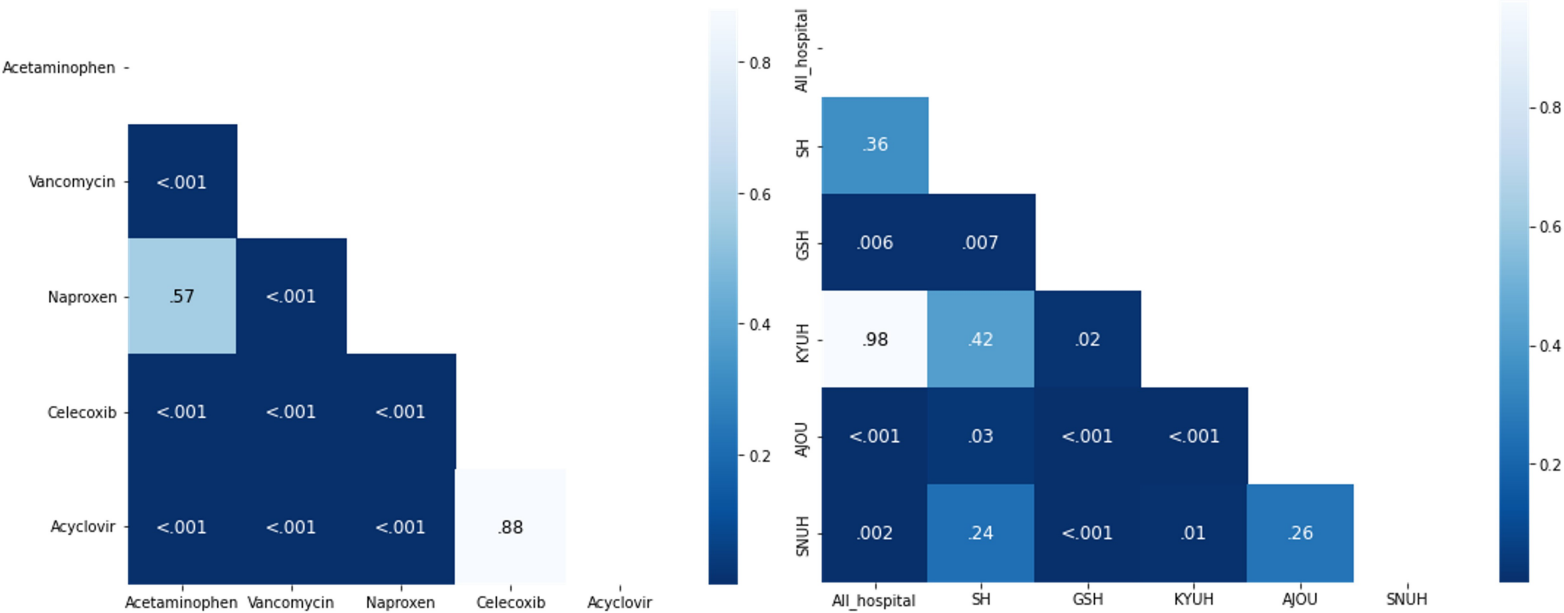
(**A**) Comparison of acute kidney injury (AKI) onset time between drugs and (**B**) AKI onset time between hospitals. The *P* values were obtained by conducting independent 2-tailed *t* tests between each aggregated pair. AJUH: Ajou University Hospital; GSH: Gangnam Severance Hospital; KYUH: Konyang University Hospital; NCC: National Cancer Center; SH: Severance Hospital; SNUH: Seoul National University Cancer Hospital.

To analyze the differences in drug patterns at the time of AKI occurrence, we assessed the pattern for each drug. The median number of days for the occurrence of AKI among the drug and cohort patients in the entire hospital was 17 (IQR 7-33 days). Vancomycin appeared after a median period of 12 days, followed by naproxen (18 days), acetaminophen (19 days), celecoxib (22 days), and acyclovir (23 days). When comparing the IQR values, celecoxib (10‐41 days) and Acyclovir (10‐41 days) showed a relatively broad distribution, whereas acetaminophen (9‐34 days) and naproxen (8‐34 days) were distributed over 25 days. Vancomycin (5‐25 days) exhibited the narrowest distribution. Celecoxib and acyclovir tended to be relatively distributed compared to acetaminophen, vancomycin, and naproxen. We compared the onset times of all drug pairs and hospital pairs to check the similarity in AKI occurrence (Figure 2). The patterns between specific drugs was similar for celecoxib and acyclovir (*P*=.88) and for acetaminophen and naproxen (*P*=.57). The patterns between hospitals were similar for SH and AJUH (*P*=.98), SH and GSH (*P*=.36), GSH and AJUH (*P*=.42), GSH and NCC (*P*=.24), and SNUH and NCC (*P*=.26).

### AKI Prediction Model Performance

**Figure 3.**
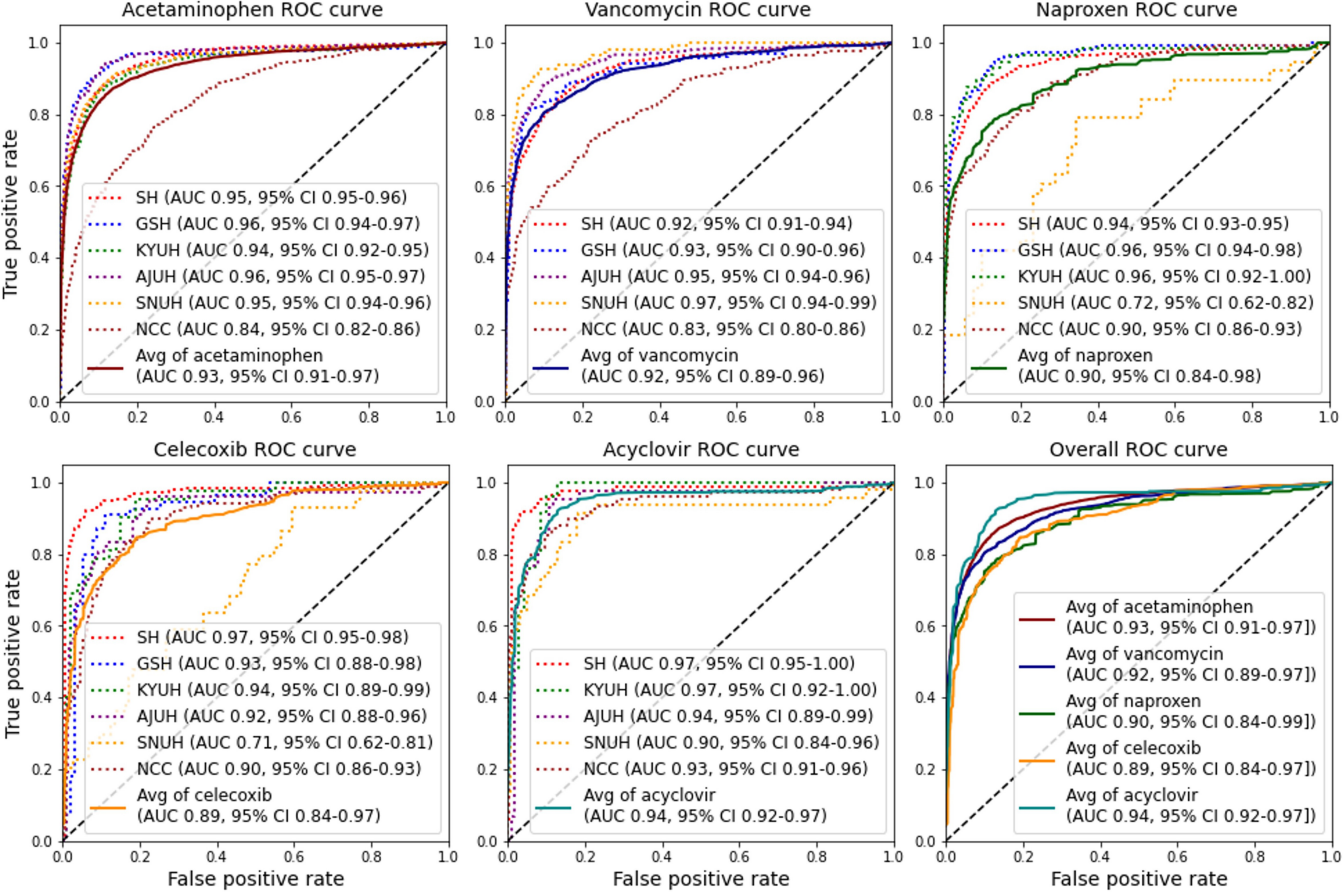
Receiver operating characteristic (ROC) curves and areas under the curve (AUCs) of the acute kidney injury prediction model for each hospital and each drug. The square brackets indicate the 95% CI. AJUH: Ajou University Hospital; GSH: Gangnam Severance Hospital; KYUH: Konyang University Hospital; NCC: National Cancer Center; SH: Severance Hospital; SNUH: Seoul National University Cancer Hospital.

The AUROC for each drug and hospital to evaluate the AKI predictive model, based on respective test sets (internal validation) is shown in Figure 3. A total of 26 trained models achieved a high AUROC value, of 0.92 on average, with each verification data set. In addition, among the averages of the drugs, acyclovir had the highest average AUROC score of 0.94, followed by acetaminophen (0.93), vancomycin (0.92), naproxen (0.90), and celecoxib (0.89). The highest AUROC value (0.97) was observed for the model of SH’s celecoxib and acyclovir, SNUH’s vancomycin, and KYUH’s acyclovir prescription patients.

Multimedia Appendix 2 presents data on the precision, accuracy, *F*_1_-score, and AUPRC of each predictive model. Overall, the average accuracy of the AKI prediction models was 0.88, whereas the average AUPRC and *F*_1_-scores were both 0.78. The acyclovir prescription model achieved the highest accuracy score (0.91), followed by vancomycin (0.90), acetaminophen (0.89), naproxen (0.89), and celecoxib (0.86). Individually, the acyclovir SH model showed the best performance, with an AUPRC of 0.92 and an accuracy of 0.91.

### Temporal Feature Importance of the AKI Prediction Model

**Figure 4.**
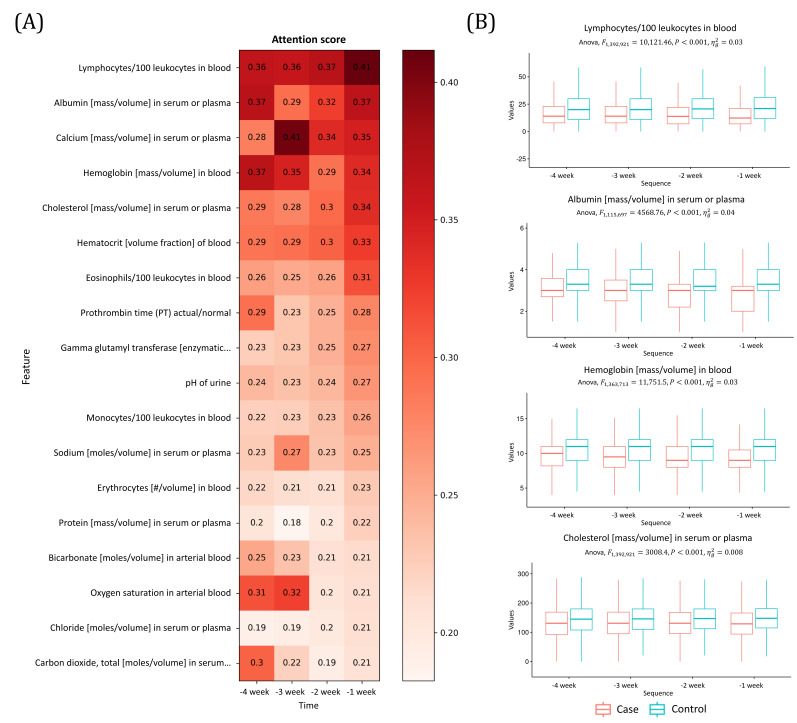
(**A**) Temporal attention score of important features of acute kidney injury (AKI) prediction model and (**B**) distribution of data over time (*P* values: repeated measures ANOVA). The figure shows the change over a 4-week period prior to the AKI event.

To interpret the AKI prediction model, we demonstrated the temporal attention values of each contributing variable in the 4 weeks prior to AKI onset, which were weighted aggregates from the model for each drug and hospital, as shown in Figure 4A. The temporal change pattern of the actual data corresponding to each variable in the 4 weeks prior to AKI onset is shown in Figure 4B. We also confirmed the difference in the distribution of highly important features between the case and control data using a 1-way ANOVA. The attention scores for all variables across all hospitals are detailed in Multimedia Appendix 3

**Figure 5.**
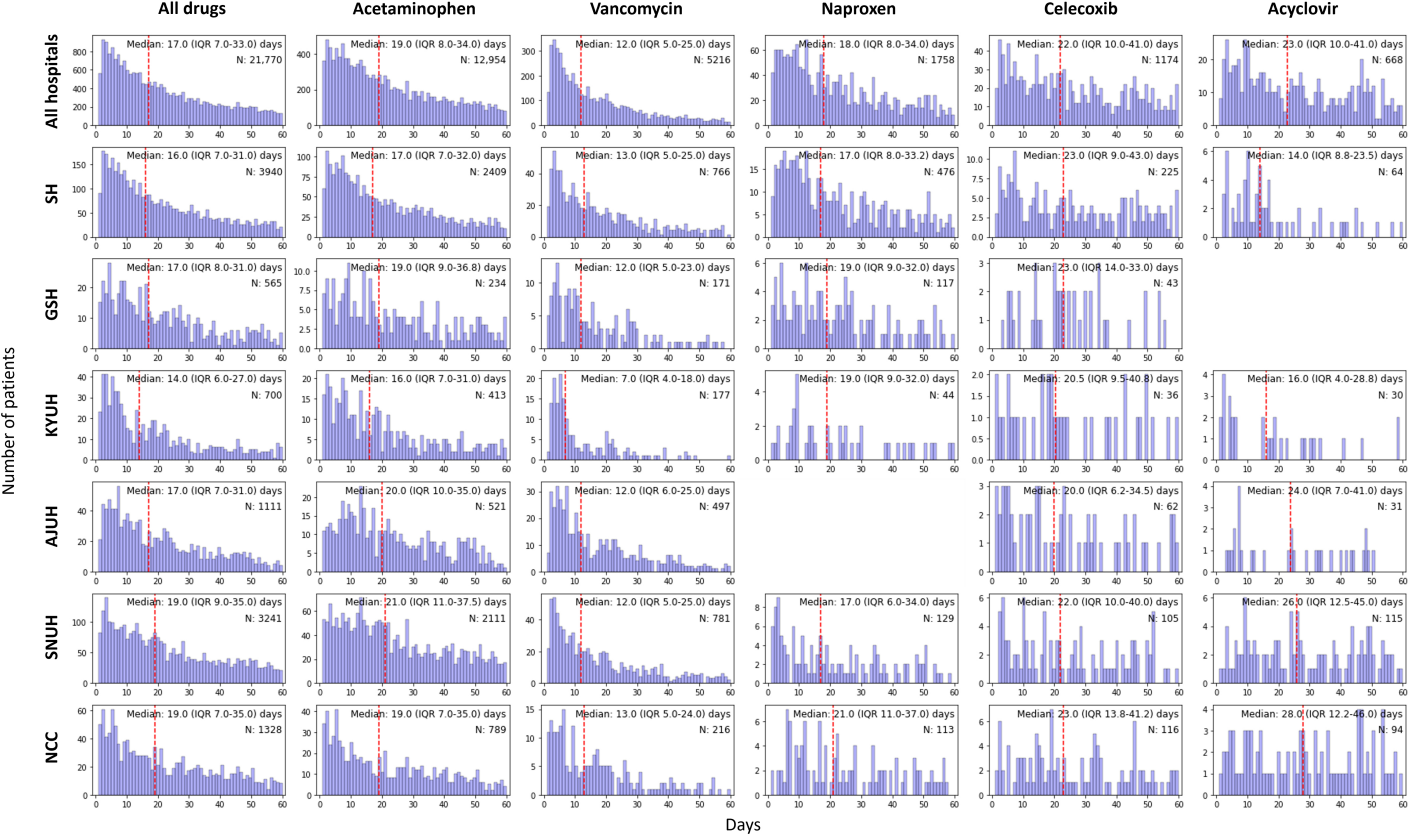
Acute kidney injury (AKI) onset time after drug administration at various medical centers. The red lines shows the median value. AJUH’s naproxen and GSH’s acyclovir cases were excluded as the number of AKI case groups was less than 20. AJUH: Ajou University Hospital; GSH: Gangnam Severance Hospital; KYUH: Konyang University Hospital; NCC: National Cancer Center; SH: Severance Hospital; SNUH: Seoul National University Cancer Hospital.

The last week of lymphocytes (attention score at −1 week: 0.41) and the second week of calcium (attention score at −3 weeks: 0.41) showed the highest attention scores, followed by albumin (attention score at −1 week: 0.37; attention score at −4 weeks: 0.37), hemoglobin (attention score at −4 weeks: 0.37), and cholesterol (attention score at −4 weeks: 0.37). In Figure 5, the distribution of data by variable between the 2 groups was confirmed using actual data. There was a difference in data distribution between the case and control groups from the beginning. The values of lymphocytes, albumin, and hemoglobin in the case group decreased over time, while urine pH and prothrombin time in the case group tended to increase over time.

## Discussion

### Principal Findings

In this study, we developed time series–based IMV-LSTM models to predict AKI in patients taking specific nephrotoxic drugs using CDM-based DRNs in a 6-hospital EHR-based system. The principal findings are as follows: first, this study provides an interpretation of the temporal importance of variables for predicting AKI, and the models also achieved high performance, with an average AUC of 0.92%. Second, our study is a scalable multicenter study using a DRN, which can contribute to understanding drug-induced AKI. To the best of our knowledge, this is the first study to build an AKI prediction model by applying a time series–based IMV-LSTM model to a CDM using EHR data from 6 hospitals.

We established a retrospective cohort of patients who took nephrotoxicity-inducing drugs at 6 hospitals. With respect to demographic characteristics, we observed variations in the overall patient count and prevalence of comorbidities when comparing individuals with AKI and without AKI across different hospitals. Nevertheless, the majority of patients who developed AKI at most hospitals were older than 60 years and had a high prevalence of comorbidities, including cancer (n=6001, 69.43%), hypertension (n=2404, 27.81%), diabetes (n=1679, 19.43%), and chronic liver disease (n=1231, 14.24%), which is consistent with findings reported in previous studies [32-35].

The pattern of each drug’s association with AKI (Figure 5) showed that the median number of days for AKI onset when using nephrotoxic drugs was 17 (IQR 7-33) days. The onset occurred earliest with vancomycin (12, IQR 5-25 days) and latest with acyclovir (23, IQR 10-41 days). In previous studies [36,37], the time to onset of vancomycin-induced AKI showed a similar pattern to our results. We also found differences in the AKI onset between different classes within the same NSAID, and the multicenter AKI cohort showed similarities between hospitals. The finding of similar patterns in the AKI onset in the multicenter cohort supports the reliability of the AKI cohort and increases the explanatory power of AKI prediction models.

AKI is common among inpatients [38,39]. Previous models predicted AKI in the intensive care unit (ICU) and in surgical patients during hospital admission. For example, Zimmerman et al [40] predicted the occurrence of AKI in ICU inpatients (AUC 0.783), and Tseng et al [41] developed a predictive score for the development of AKI after cardiac surgery (AUC 0.839). Hsu et al [42] developed a risk score function for community-acquired AKI for inpatients (AUC 0.818). Koyner et al [10] developed a model to predict AKI in hospitalized patients (AUC 0.90). Despite this progress, few studies have applied time series deep learning to provide a temporal interpretation of drug-induced AKI, and our model stands out because it can predict nephrotoxic drug–induced AKI in a diverse hospital population.

This study achieved improved performance compared to previous AKI studies using recurrent neural networks (RNNs) [21,43-45]. Our model improves performance up to an AUC of 0.97 and an overall average of 0.92, which outperforms previous studies showing AKI prediction with RNN-based methods by Kim et al [43] in hospitalized patients (AUC 0.927), Rank et al [45] in cardiac surgery patients (AUC 0.893), and Xu et al [44] in inpatients (AUC 0.908). These results show promise for our model as a tool to predict AKI and facilitate early intervention and mitigation strategies for patients.

This study also provides additional interpretations regarding the temporal importance of features for AKI prediction. Some studies have reported information on interpretability or provided information about the interpretability of variables at the feature importance level [46]. However, our results show the importance of variables and the temporal importance of variables in the development of AKI. In this study, we highlight the vital role of temporal patterns of various indicators, such as lymphocytes, calcium, albumin, hemoglobin, and cholesterol, in predicting disease states, particularly the onset of AKI. The temporal pattern of lymphocytes increased gradually, peaking 1 week before AKI onset. The use of lymphocyte and neutrophil counts as predictive factors for AKI is consistent with other studies [47,48]. Calcium shows a pattern of peaking 3 weeks before AKI onset, and Prior studies showed an association between impacted calcium metabolism and AKI [49,50]. Albumin shows the highest pattern 1 and 4 weeks before onset, and low serum albumin levels (hypoalbuminemia) are a predictor of AKI [48,51,52]. Hemoglobin shows a pattern with a peak 4 weeks before onset, and previous studies have shown that the risk of AKI increases stepwise with a further decrease in hemoglobin concentration [53]. Temporal variations in variables based on reported laboratory data for the early detection of AKI emphasize the importance of monitoring and early intervention in populations.

In addition, this retrospective study can be followed by a subsequent study to validate the practicality of the AKI prediction model in clinical practice by applying it to a prediction system in a hospital EHR.

### Limitations

This study has several limitations. First, because we used the CDM, it does not reflect the full range of clinical data. For example, we could not include admission records, which would have revealed a patient’s condition. However, the use of CDM data allowed for a multicenter study that could be easily extended to other institutions that have converted to the CDM. Second, this was a retrospective study and could not address the underlying causes of AKI. Therefore, prospective studies are needed for validation with actual clinical data. Third, as with all retrospective studies, there may be unintentional patient selection bias and unaccounted-for confounders. However, to compensate for these limitations, we tried to equalize the distribution of patient characteristics through PSM. We also used a limited follow-up period to minimize the impact of these factors.

### Conclusions

This study demonstrates the high performance of the IMV-LSTM method for AKI prediction using hospital EHR-based time series data. Our model can provide real-time assessment of AKI occurrence and individualized risk factors for AKI using time series data. We also demonstrated the robustness of our model through multicenter validation using a CDM through a DRN of 6 hospitals in South Korea, which also proves that scalability to other institutions that are converted to the CDM is possible. This may provide an objective quantitative tool for identifying patients at risk of developing AKI.

## Supplementary material

10.2196/47693 Multimedia Appendix 1 Demographics for each institution.

10.2196/47693 Multimedia Appendix 2 Model performance.

10.2196/47693 Multimedia Appendix 3 Feature importance.

## Abbreviations

ADR: adverse drug reaction
AI: artificial intelligence
AJUH: Ajou University Hospital
AKI: acute kidney injury
AUPRC: area under the precision-recall curve
AUROC: area under the receiver operating characteristic curve
CDM: common data model
DRN: distributed research network
EHR: electronic health record
FDA: Food and Drug Administration
GSH: Gangnam Severance Hospital
ICU: intensive care unit
IMV-LSTM: interpretable multivariable long short-term memory
KDIGO: Kidney Disease Improving Global Outcomes
KYUH: Konyang University Hospital
MIMIC: Medical Information Mart for Intensive Care
NCC: National Cancer Center
NSAID: nonsteroidal anti-inflammatory drug
OMOP-CDM: Observational Medical Outcomes Partnership Common Data Model
PSM: propensity score matching
RNN: recurrent neural network
SCr: serum creatinine
SH: Severance Hospital
SNUH: Seoul National University Cancer Hospital
ULN: upper limit of normal
